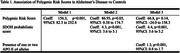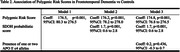# Predicting Neurodegenerative Diseases: Unveiling the Interplay of Genetics and Social Determinants

**DOI:** 10.1002/alz.089721

**Published:** 2025-01-09

**Authors:** Stefanie Danielle Pina Escudero, Joaquín Migeot, Hernan Hernandez, Juliana Acosta‐Uribe, J. Nicholas Cochran, Jared W Taylor, José Alberto Ávila Funes, Diana Matallana, Francisco Lopera, Nilton Custodio, María Isabel Behrens, Andrea Slachevsky Chonchol, Martin Alejandro Bruno, Luis Ignacio Brusco, Leonel Tadao Takada, Elisa de Paula, França Resende, Pablo A Reyes, Nancy Gelvez, Teresita Ramos Franco, Bárbara Bruna, Daniela P Ponce, Dafne Estefania Durón, Rosa Montesinos, Maria Beatriz Bistue Millon, Marcelo Adrian Maito, Shireen Javandel, Maria Eugenia Godoy, Dan Vitale, Caroline Pantazis, Mike A Nalls, Andrew Singleton, Bruce L. Miller, Agustin Ibañez, Kenneth S. Kosik, Jennifer S. Yokoyama, Multi‐Partner Consortium to Expand Dementia Research in Latin America (ReDLat)

**Affiliations:** ^1^ University of California, San Francisco, San Francisco, CA USA; ^2^ Latin American Brain Health Institute (BrainLat), Universidad Adolfo Ibáñez, Santiago Chile; ^3^ Latin American Brain Health Institute (BrainLat), Universidad Adolfo Ibañez, Santiago Chile; ^4^ Grupo de Neurociencias de Antioquia, Universidad de Antioquia, Medellin, Medellin Colombia; ^5^ University of California Santa Barbara, Santa Barbara, CA USA; ^6^ HudsonAlpha Institute for Biotechnology, Huntsville, AL USA; ^7^ Instituto Nacional de Ciencias Médicas y Nutrición Salvador Zubirán, Mexico City, DF Mexico; ^8^ Pontificia Universidad Javeriana, Bogota, Cundinamarca Colombia; ^9^ Hospital Universitario Fundación Santa Fe, Bogotá Colombia; ^10^ Hospital Universitario San Ignacio, Bogotá Colombia; ^11^ Neurosciences Group of Antioquia, University of Antioquia, Medellin Colombia; ^12^ Grupo de Neurociencias de Antioquia, Facultad de Medicina, Universidad de Antioquia, Medellín Colombia; ^13^ Neurosciences Group of Antioquia, University of Antioquia, Medellín Colombia; ^14^ Research unit, Instituto Peruano de Neurociencias, Lima Peru; ^15^ Instituto Peruano de Neurociencias, Lima, Lima Peru; ^16^ Centro de Investigación Clínica Avanza (CICA), Hospital Clínico Universidad de Chile, Santiago Chile; ^17^ Hospital Clínico de la Universidad de Chile, Santiago de Chile Chile; ^18^ Clínica Alemana‐Universidad del Desarrollo, Santiago Chile; ^19^ Neuropsychology and Clinical Neuroscience Laboratory (LANNEC), Physiopathology Department ‐ ICBM, Neuroscience and East Neuroscience Departments, Faculty of Medicine, Universidad de Chile, Santiago Chile; ^20^ Geroscience Center for Brain Health and Metabolism (GERO), Santiago Chile; ^21^ Memory and Neuropsychiatric Center (CMYN), Neurology Department, Hospital del Salvador and Faculty of Medicine, Universidad de Chile, Santiago Chile; ^22^ Instituto de Ciencias Biomédicas (ICBM) Facultad de Ciencias Médicas, Universidad Catoóica de Cuyo, San Juan Argentina; ^23^ McGill University, Montreal, QC Canada; ^24^ ReDLat, San Juan Argentina; ^25^ ALZAR ‐ Argentine Alzheimer's Association, Buenos Aires, CABA Argentina; ^26^ National Scientific and Technical Research Council (CONICET), Buenos Aires Argentina; ^27^ Universidad de Buenos Aires, Buenos Aires Argentina; ^28^ Hospital das Clínicas, University of Sao Paulo Medical School, São Paulo Brazil; ^29^ Faculdade de Medicina de Ciências Médicas de Minas Gerais, Belo Horizonte Brazil; ^30^ Global Brain Health Institute, University of California, San Francisco, CA USA; ^31^ Hospital del Salvador & Faculty of Medicine, University of Chile., Santiago Chile; ^32^ Instituto Nacional de Ciencias Médicas y Nutrición Salvador Zubirán, Ciudad de México, DF Mexico; ^33^ Unit Cognitive Impairment and Dementia Prevention, Peruvian Institute of Neurosciences, Lima, Peru, Lima, Lima Peru; ^34^ Universidad Católica de Cuyo / CONICET, San Juan Argentina; ^35^ Instituto de Ciencias Biomédicas (ICBM), Facultad de Ciencias Médicas, Universidad Católica de Cuyo, San Juan Argentina; ^36^ Universidad de San Andres, Buenos Aires Argentina; ^37^ Data Tecnica International, Washington, DC USA; ^38^ National Institute on Aging, Bethesda, MD USA; ^39^ Center for Alzheimer's and Related Dementias, National Institute on Aging and National Institute of Neurological Disorders and Stroke, National Institutes of Health, Bethesda, MD USA; ^40^ Global Brain Health Institute, San Francisco, CA USA; ^41^ Memory and Aging Center, UCSF Weill Institute for Neurosciences, University of California, San Francisco, San Francisco, CA USA; ^42^ Latin American Brain Health (BrainLat) Institute, Universidad Adolfo Ibáñez, Peñalolén, Santiago Chile; ^43^ Global Brain Health Institute (GBHI), Trinity College Dublin (TCD), Dublin, Dublin Ireland; ^44^ Global Brain Health Institute, University of California San Francisco, San Francisco, CA USA

## Abstract

**Background:**

Predicting Alzheimer's disease (AD) and frontotemporal dementia (FTD) using polygenic risk scores (PRS) for late‐onset forms holds promise, but its accuracy might be influenced by social determinants of health (SDOH). This study explores how considering SDOH alongside genes can improve prediction, focusing on potential differences for each disease.

**Methods:**

Employing logistic regression in 677 individuals (287 AD, 102 FTD, and 288 controls) aged 40‐80 from the ReDLat study across six Latin American countries, we investigated the potential for SDOH to modify the association between PRS and susceptibility to AD and FTD. Analyses were adjusted for a probabilistic score derived from models comparing disease groups to controls with SDOH data (education, occupation, economic stability, healthcare access and quality, and social context) and APOE ε4 carrier status to account for confounding effects.

**Results:**

Although univariate association tests revealed robust links between PRS and both diseases, adjusted models presented a nuanced picture. In AD, the SDOH score and APOE ε4 carrier status significantly attenuated the PRS effect (p=0.14), suggesting these factors modify genetic risk. In FTD, however, SDOH did not influence the PRS contribution. These findings highlight the potentially distinct roles of social factors in different neurodegenerative pathways.

**Conclusion:**

The significant modification of PRS effects in AD by SDOH and APOE ε4 underscores the need for comprehensive approaches in future research and interventions in Latin America. Conversely, the unaltered PRS contribution in FTD emphasizes distinct intricacies in gene‐environment interactions. These findings necessitate considering both realms in future efforts, paving the way for targeted strategies in AD and FTD prevention and treatment.